# PGC1-Alpha/Sirt3 Signaling Pathway Mediates the Anti-Pulmonary Fibrosis Effect of Hirudin by Inhibiting Fibroblast Senescence

**DOI:** 10.3390/biomedicines12071436

**Published:** 2024-06-27

**Authors:** Bin He, Qian Zeng, Yumei Tian, Yuyang Luo, Minlin Liao, Wenjie Huang, Bin Wu, Ziqiang Luo, Xiaoting Huang, Wei Liu, Siyuan Tang

**Affiliations:** 1 School of Nursing, Hunan University of Medicine, Huaihua 418000, China; hebin2008082022@163.com (B.H.); moldrains@163.com (Y.T.); hnyyxyhwj@163.com (W.H.); meiliang692021@163.com (B.W.); 2 Xiangya Nursing School, Central South University, Changsha 410013, China; zengqian02@csu.edu.cn (Q.Z.); 227811043@csu.edu.cn (Y.L.); liaominlin120@csu.edu.cn (M.L.); huangxiaoting@csu.edu.cn (X.H.); 3 Xiangya School of Medicine, Central South University, Changsha 410013, China; luoziqiang@csu.edu.cn

**Keywords:** idiopathic pulmonary fibrosis, hirudin, fibroblast senescence, PGC1-alpha/Sirt3 pathway

## Abstract

Idiopathic pulmonary fibrosis (IPF) is a chronic, progressive fibrotic lung disease for which there is a lack of effective pharmacological treatments. Hirudin, a natural peptide extracted from leeches, has been used for broad pharmacological purposes. In this study, we investigated the therapeutic effects of hirudin on IPF and its related mechanism of action. By constructing a mouse model of pulmonary fibrosis and treating it with hirudin in vivo, we found that hirudin exerted anti-fibrotic, anti-oxidative, and anti-fibroblast senescence effects. Moreover, using an in vitro model of stress-induced premature senescence in primary mouse lung fibroblasts and treating with hirudin, we observed inhibition of fibroblast senescence and upregulation of PGC1-alpha and Sirt3 expression. However, specific silencing of PGC1-alpha or Sirt3 suppressed the anti-fibroblast senescence effect of hirudin. Thus, the PGC1-alpha/Sirt3 pathway mediates the anti-fibroblast senescence effect of hirudin, potentially serving as a molecular mechanism underlying its anti-fibrosis and anti-oxidative stress effects exerted on the lungs.

## 1. Introduction

Idiopathic pulmonary fibrosis (IPF) is a chronic, progressive, and fatal interstitial lung disease characterized by inflammatory cell infiltration in the alveolar interstitium, massive proliferation of fibroblasts, and excessive deposition of extracellular matrix (ECM) proteins. These changes ultimately destroy the pulmonary tissue structure [1]. IPF morbidity and mortality rates have increased globally in recent years, and currently, the only definitive treatment is lung transplantation [2]. Once diagnosed with IPF, patients frequently have multiple comorbidities and a shorter life expectancy [3]. Therefore, it is imperative to develop drugs for specific treatment.

IPF is an age-related disease that commonly occurs in older individuals [4,5]. Although the specific etiology and pathogenesis of IPF remain unknown, researchers have revealed that the senescence of various key cells in lung tissue, including fibroblasts, significantly contributes to the development of pulmonary fibrosis [6]. Fibroblasts play an important role in repairing localized microinjuries in lung tissue during the onset and progression of IPF [7]. Senescent fibroblasts persist in the lung tissues of those with pulmonary fibrosis diseases [8,9,10], and these senescent fibroblasts are usually accompanied with cell cycle inhibition, telomere shortening, senescence-associated secretory phenotypes (SASPs), and mitochondrial dysfunction, which are closely associated with IPF development [11,12]. In addition, inhibition of fibroblast senescence can effectively ameliorate pulmonary fibrosis in mice [13,14,15]. To inhibit IPF progression caused by fibroblast senescence, several protein molecules, including those of the sirtuin family, have been identified as significant players in anti-aging. Among them, Sirt3 has been demonstrated to exert an anti-pulmonary fibrosis effect by inhibiting fibroblast senescence. The detailed mechanisms by which Sirt3 exerts its anti-aging effects are currently unknown; the PGC1-alpha/Sirt3 pathway has been shown to play an important role in the body’s regulation of oxidative stress, mitochondrial function, and cellular senescence, and can also play a role in inhibiting fibrosis through activation of the PGC1-alpha/Sirt3 pathway [16]. Therefore, the development of therapeutic agents that target fibroblast senescence demonstrates good feasibility and promising prospects.

Hirudin, a peptide first extracted from the salivary glands of leeches, is a natural thrombin inhibitor, with anti-fibrotic, anti-thrombotic, anti-injury, and anti-tumor properties [17]. Coagulation activation in response to epithelial injury plays a crucial part in the progression of IPF [18,19]. As a key molecule in the coagulation process, thrombin promotes the proliferative activities of fibrinogen on fibroblasts and can also induce fibrosis-related cytokines [20,21,22]. Since the role of thrombin in promoting the development of pulmonary fibrosis has been suggested, and significant therapeutic effects have been demonstrated by several thrombin inhibitors on IPF [23], we hypothesized that hirudin, with its potent thrombin-inhibiting ability, would also exert an anti-pulmonary fibrosis effect. Evidence suggests that hirudin has a protective effect against acute lung injury [24]. Moreover, a new fusion protein, rhSOD2–hirudin, produced by fusing hirudin with human manganese superoxide dismutase (hSOD2), has been shown to have therapeutic effects against pulmonary fibrosis [25]. However, no relevant studies exploring the direct anti-pulmonary fibrosis effects of hirudin have been previously conducted. Therefore, we aim to explore the direct anti-pulmonary fibrosis effect of hirudin and its mechanism of action in an effort to improve our understanding of hirudin’s pharmacological properties and mechanisms. This could offer a novel option for the pharmacological management of IPF.

## 2. Materials and Methods

### 2.1. Animal Protocols

C57BL/6 mice (male; eight-week-old) were housed in a specific pathogen-free environment at the Central South University Department of Zoology. A total of 10 mice out of 110 were randomly selected as controls, while the remaining mice were intratracheally injected with 50 μL bleomycin (3 mg/kg; Nippon Kayaku, Tokyo, Japan) to construct a pulmonary fibrosis model. Control mice received intraperitoneal injections of the solvent from days 15 to 28 following tracheal injection of 50 μL saline. On day 14 after bleomycin administration in the mice used for modeling, they were stratified by body weight and divided into four groups, lung fibrosis group, high-, medium-, and low-dose groups, each with an equal number of mice. From day 15 to 28, mice in each group were intraperitoneally administered with the solvent, hirudin at doses of 10 mg/kg (Macklin, Shanghai, China), hirudin at doses of 3 mg/kg, and hirudin at doses of 1 mg/kg, respectively. 

### 2.2. Masson Staining, Hematoxylin–Eosin Staining, and Ascroft Scoring

The lung tissues were fixed and embedded in paraffin wax prior to sectioning. The sections were then deparaffinization and stained with hematoxylin and eosin (HE; Pinuofei Biological, Wuhan, China) and Masson’s trichrome (Pinuofei Biological), dehydrated, and sealed. Three researchers independently evaluated fibrosis severity in each HE-stained section using the Ashcroft scoring method, and the final results were averaged from their scores [26].

### 2.3. Determination of Hydroxyproline in Lung Tissue

In accordance with the instructions provided by the hydroxyproline assay test kit (Nanjing Jiancheng Biotechnology Institute, Nanjing, China), we prepared a tissue homogenate for the lung and added a variety of reagents. Subsequently, the homogenate was allowed to stand at room temperature, the supernatant was centrifuged, and the absorbance value was measured at 550 nm.

### 2.4. Immunofluorescence Staining of Lung Tissue

The TSA immunofluorescence kit was obtained from Aifang Biological (Changsha, China). Paraffin sections underwent sequential processing steps including dewaxing, antigen repair, endogenous peroxidase blocking, non-specific antigen sealing, and overnight incubation with anti-P21 antibody (Proteintech, Wuhan, China), followed by horseradish peroxidase (HRP) secondary antibody incubation, and subsequent incubation with TSA fluorescent dye. Following the same procedure, sections were treated with an anti-α-SMA antibody (Proteintech), and the nuclei were stained with 4′,6-diamidino-2-phenylindole (DAPI) dye.

### 2.5. Reactive Oxygen Species Staining of Lung Tissue

The frozen sections were heated to room temperature, the tissue was circled with an immunohistochemical pen, and a tissue anti-fluorescence quencher was added. The circled tissue was incubated with drops of reactive oxygen species (ROS) stain, while the nuclei were stained with DAPI dye, and the sections were then sealed. Images were captured using fluorescence microscopy.

### 2.6. Determination of Malondialdehyde and Glutathione Contents and Total Superoxide Dismutase Activity in Lung Tissue

Total superoxide dismutase (SOD) activity and malondialdehyde (MDA) and glutathione (GSH) contents were determined using SOD, MDA, and GSH assay kits, respectively (Nanjing Jianjian Biotechnology Institute, Nanjing, China). The mouse lung tissue was homogenized according to the manufacturer’s instructions, followed by the sequential addition of each reagent in the kits. The corresponding absorbance values were measured, and the total SOD activity and MDA and GSH contents were calculated according to the absorbance values.

### 2.7. RNA Extraction and Real-Time Quantitative Polymerase Chain Reaction

Total RNA was isolated from lung tissues or cells using TRIzol (Thermo Fisher Scientific, MA, USA), followed by being reversed to cDNA. The primers used in this experiment were ordered from Sangon Biotech (Shanghai, China), and their sequences are shown in Table 1.

### 2.8. Western Blot

Total proteins were extracted from mouse lung tissues or cells using radioimmunoprecipitation lysates containing phosphatase inhibitors, protease inhibitors, and phenylmethylsulfonyl fluoride (Solarbio, Beijing, China). Gel electrophoresis was then performed on the proteins, followed by membrane transference to a polyvinylidene fluoride. Subsequently, the membranes were blocked with 5% skimmed milk for 2 h and incubated overnight at 4 °C with antibodies against β-actin, α-SMA, collagen I, p21, p16, PGC1-alpha, and Sirt3 (β-actin, α-SMA, PGC1-alpha, and Sirt3 obtained from Proteintech, and collagen I, p21, and p16 obtained from Abcam, Cambridge, UK). Following primary antibody incubation, the membranes were treated with the respective secondary antibody (Proteintech) for 2 h. Finally, protein expression was observed using an enhanced chemiluminescence developer.

### 2.9. Cell Extraction and Cell Culture

Primary mouse lung fibroblasts were extracted following previously described methods [27,28]. Briefly, mice were euthanized, and lung tissues were minced. The minced tissues were then placed in a solution of type I collagenase (1 mg/mL; Gibco, Waltham, MA, USA) and incubated in a 37 °C water bath for 1 h. After centrifugation, the cells were filtered, resuspended several times, and then seeded in DMEM–high glucose (Procell, Wuhan, China) containing 20% fetal bovine serum (Procell) and 1% penicillin–streptomycin solution (Procell). Cells were cultured in a humidified incubator at 37 °C with 5% CO_2_.

### 2.10. Hydrogen Peroxide-Induced Senescence of Fibroblasts

Fibroblasts were incubated with a medium containing 200 µΜ hydrogen peroxide (Sigma, St. Louis, MO, USA) for 2 h after seeding the plate. Subsequently, cells were washed three times with PBS, and then the fibroblasts were incubated with a complete medium for 48 h to induce cellular senescence.

### 2.11. Determination of Cell Viability

Fibroblasts were inoculated in 96-well plates and co-incubated with different concentrations of hirudin for 48 h. After rinsing with PBS, a reagent (Elabscience, Wuhan, China) for the Cell Counting Kit-8 (CCK-8) was added, and then cells were incubated at 37 °C. Lastly, we measured the absorbance of each well at 450 nm.

### 2.12. Cellular Immunofluorescence

Following the instructions of the TSA fluorescence kit, cells were fixed with 4% paraformaldehyde, permeabilized, and then blocked for non-specific antigen binding. They were then incubated with a P21 antibody (Proteintech) overnight, followed by secondary antibodies conjugated with HRP. Nuclei were stained with DAPI dye, and fluorescence images were captured using a fluorescence microscope.

### 2.13. Cellular ROS Staining

Fibroblasts were treated at 37 °C with an ROS fluorescent probe (Beyotime Biotechnology, Shanghai, China), followed by two rounds of DMEM washing. Subsequently, the cells were examined using a fluorescence microscope, and images were captured.

### 2.14. siRNA-Specific Gene Silencing

Transfection was performed after cell density reached 50%. The transfection reagent was mixed with siRNA (Reebok, Guangzhou, China) to form a transfection complex, incubated for 15 min, and then mixed in a full culture base without penicillin–streptomycin solution. Cells were treated accordingly after transfection.

### 2.15. Statistical Analysis

We used GraphPad Prism 8.3.1 for statistical analyses. All data are summarized as the mean ± standard deviation. Comparisons between multiple groups were performed using one-way analysis of variance and Tukey’s multiple comparison tests. A *p*-value less than 0.05 presented statistical significance.

## 3. Results

### 3.1. Hirudin Inhibited Bleomycin-Induced Pulmonary Fibrosis in Mice

**Figure 1:**
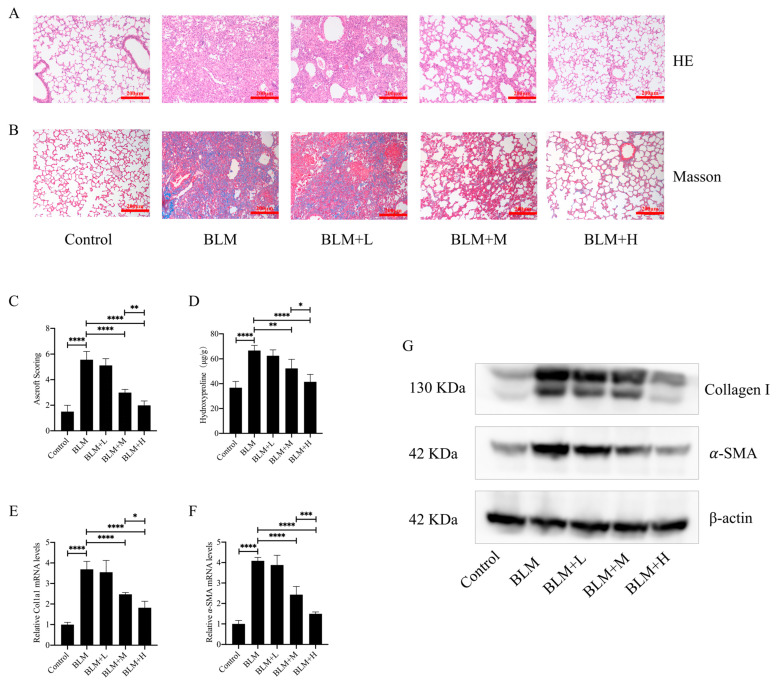
Hirudin has a therapeutic effect in bleomycin-induced lung fibrosis in mice. Bleomycin tracheal injection was used to create a pulmonary fibrosis model. From days 15 to 28, intraperitoneal injections of hirudin at high, moderate, and low doses were then administered. Histopathological structure and ECM deposition changes in mouse lung tissue (**A**,**B**). The overall fibrosis of lung tissue in groups of mice (**C**). The level of hydroxyproline in mouse lung tissue (**D**). The mRNA expression levels of *Col1a1* and *ACTA2* (**E**,**F**). The amount of type I collagen and α-SMA in mouse lung tissues (**G**). BLM + H, 10 mg/kg group; BLM + M, 3 mg/kg group; BLM + L, 1 mg/kg group; BLM, pulmonary fibrosis model group; and Control, control group. All data are expressed in the mean ± standard deviation; *n* = 8, * *p* < 0.05; ** *p* < 0.01; *** *p* < 0.001; and **** *p* < 0.0001.

To investigate the direct anti-pulmonary fibrosis effect of hirudin, we constructed a pulmonary fibrosis model by injecting bleomycin into the trachea of mice. From days 15 to 28 after bleomycin injection, we administered different doses of hirudin intraperitoneally. The staining results demonstrated that hirudin notably improved the disorganization of the lung tissue structure in mice with pulmonary fibrosis and reduced ECM deposition (Figure 1A,B). Similarly, Ascroft scores and hydroxyproline assays indicated that hirudin effectively improved the pathological changes in the lungs of these mice (Figure 1C,D). The fibrosis markers type I collagen and α-SMA have lower levels of mRNA and protein expression when exposed to hirudin, as shown by real-time quantitative polymerase chain reaction (Q-PCR) and Western blot (WB) analyses (Figure 1E–G). These findings indicate that moderate and high doses of hirudin were effective in reducing bleomycin-induced pulmonary fibrosis, with the high doses having a more significant effect than the medium doses.

### 3.2. Hirudin Reduced the Level of Oxidative Stress in Lung Tissue of Pulmonary Fibrosis Mice

**Figure 2:**
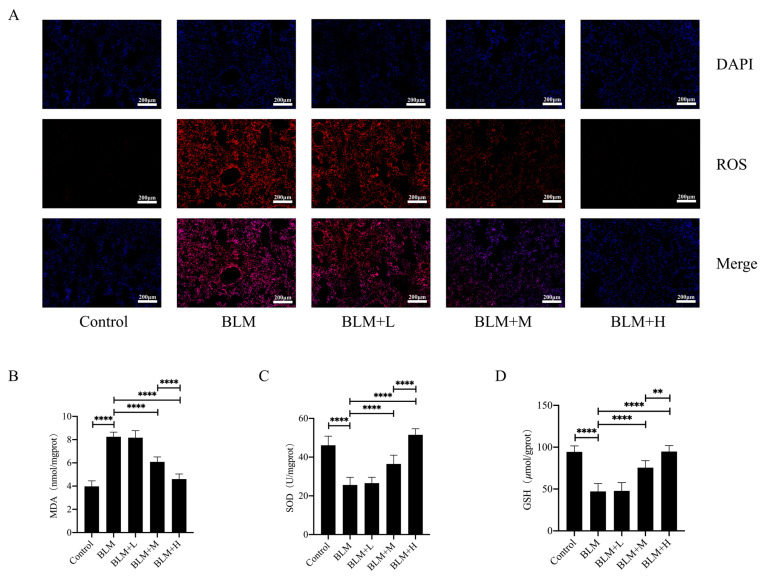
Hirudin inhibits oxidative stress in bleomycin-induced lung fibrosis. The levels of ROS in lung tissues (**A**).The MDA levels, GSH levels, and total SOD levels in lung tissues (**B**–**D**). BLM + H, 10 mg/kg group; BLM + M, 3 mg/kg group; BLM + L, 1 mg/kg group; BLM, pulmonary fibrosis model group; and Control, control group. All data are expressed in the mean ± standard deviation; *n* = 8; ** *p* < 0.01; and **** *p* < 0.0001.

rhSOD2–Hirudin has been shown to exhibit anti-oxidant effects [25]. We examined ROS levels in the lung tissues of various groups of mice using DHE staining to explore whether hirudin exerted an anti-oxidative stress effect. Moderate and high dosages of hirudin significantly reduced ROS levels in the lung tissues of mice with lung fibrosis (Figure 2A). Furthermore, we examined the levels of the oxidative stress markers GSH and MDA, as well as total SOD activity in the lung tissues of mice. We found that hirudin effectively reduced the MDA levels and increased the GSH levels and total SOD activity in the lung tissues (Figure 2B–D). These results indicate that hirudin also exhibits an anti-oxidative stress effect, with the effect of high doses of hirudin being more significant than that of the medium dose. Additionally, the anti-pulmonary fibrosis effect of hirudin may also be related to its anti-oxidative stress effect.

### 3.3. Hirudin Ameliorated Fibroblast Senescence in Lung Tissue of Mice with Pulmonary Fibrosis

**Figure 3:**
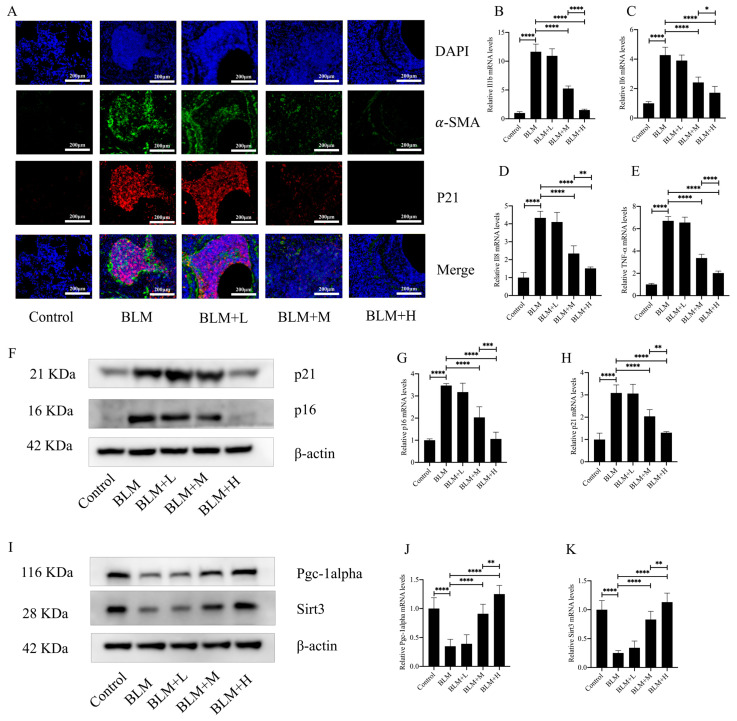
Hirudin inhibits fibroblast senescence in bleomycin-induced lung fibrosis. The levels of fibrosis and fibroblast senescence in mouse lung tissues (**A**). The mRNA expression levels of *IL1b*, *IL6*, *IL8*, and *TNF-α* in mouse lung tissues (**B**–**E**). The protein expression levels of p21, p16, PGC1-alpha, and Sirt3 in mouse lung tissues (**F**,**I**). The mRNA expression levels of *p21* and *p16* in mouse lung tissues (**G**,**H**). The mRNA expression levels of *PGC1-alpha* and *Sirt3* in mouse lung tissues (**J**,**K**). BLM + H, 10 mg/kg group; BLM + M, 3 mg/kg group; BLM + L, 1 mg/kg group; BLM, pulmonary fibrosis model group; and Control, control group. All data are expressed in the mean ± standard deviation; *n* = 8; **** *p* < 0.0001; *** *p* < 0.001; ** *p* < 0.01; and * *p* < 0.05.

Oxidative stress is an integral part of the pathological process of cellular senescence. Fibroblast senescence is a pivotal pathological mechanism in the development of IPF [29]. In the above results, we found that hirudin plays an anti-oxidative stress role while exerting an anti-pulmonary fibrosis effect. To investigate whether hirudin also plays an anti-fibroblast senescence role, we performed immunofluorescence of mouse lung tissues. The results showed that the expression of p21, a marker of cellular senescence, was similarly high in the region of α-SMA high expression. Hirudin at a medium and high dose significantly reduced the expression of α-SMA in the lung tissues of mice with pulmonary fibrosis while also reducing the expression of p21 (Figure 3A). The secretion of SASPs increases significantly during cellular senescence. Four SASPs (IL-β, IL6, IL-8, and TNF-α) were examined for expression levels in mouse lung tissues; we found that medium and high dosages of hirudin considerably decreased the levels of these SASPs (Figure 3B–E). Additionally, we examined the expression levels of the cellular senescence indicators p21 and p16. The results showed that hirudin significantly reduced the expression levels of p21 and p16 in the lung tissues of mice with pulmonary fibrosis (Figure 3F–H). This suggests that high and medium doses of hirudin significantly inhibited fibroblast senescence during the development of pulmonary fibrosis, which may be a mechanism underlying the anti-pulmonary fibrosis effect of hirudin. The PGC1-alpha/Sirt3 pathway plays an important role in anti-aging and anti-fibrotic processes [30,31]. To investigate whether the role of hirudin in fibroblast senescence is related to the activation of the PGC1-alpha/Sirt3 pathway, we examined the expression levels of PGC1-alpha and Sirt3 in lung tissues. We observed that hirudin significantly raised the expression levels of PGC1-alpha and Sirt3 in pulmonary fibrosis mouse lung tissues (Figure 3I–K). In conclusion, the anti-fibrotic effect of hirudin on pulmonary fibrosis may be related to the activation of the PGC1-alpha/Sirt3 pathway and its role in anti-fibroblast senescence.

### 3.4. Hirudin Inhibited Hydrogen Peroxide-Induced Fibroblast Senescence

**Figure 4:**
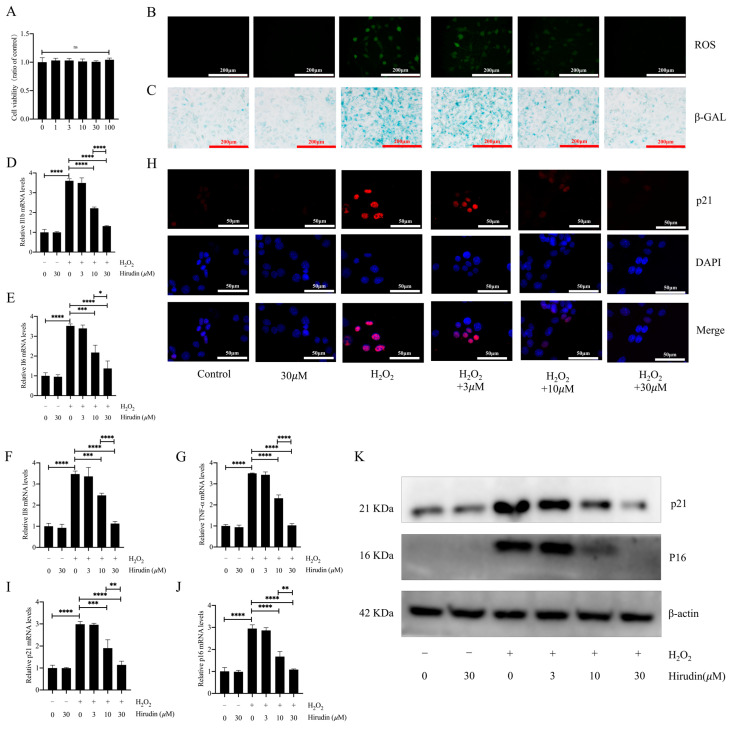
Hirudin has an inhibitory effect on hydrogen peroxide-induced senescence in fibroblasts. The effect of hirudin on the viability of fibroblasts (**A**). The level of ROS in fibroblasts (**B**). The β-galactosidase levels in fibroblasts (**C**). The mRNA expression levels of *IL1b*, *IL6*, *IL8*, and *TNF-α* in fibroblasts (**D**–**G**). The mRNA expression levels of *p21* and *p16* in fibroblasts (**I**,**J**). The expression of p21 in fibroblasts (**H**). The protein expression levels of p21 and p16 in fibroblasts (**K**). All data are presented as mean ± standard deviation, and all experiments were conducted independently and repeated a minimum of three times. * *p* < 0.05; ** *p* < 0.01; *** *p* < 0.001; and **** *p* < 0.0001.

To further investigate the anti-fibroblast senescence effects of hirudin, we induced senescence in mouse primary lung fibroblasts using hydrogen peroxide and treated them with different concentrations of hirudin to observe its effect on senescence markers. We found that none of the cell viability of fibroblasts was significantly affected by hirudin at 0–100 µΜ (Figure 4A). During cellular senescence, ROS levels typically increase. We further investigated hirudin’s effect on ROS levels during senescence of primary lung fibroblasts given our previous findings of its significant reduction of ROS levels in mice with pulmonary fibrosis. Our results demonstrated that hirudin at concentrations of 10 µM and 30 µM notably decreased ROS levels (Figure 4B). By performing β-galactosidase staining, we observed that 10 µΜ and 30 µΜ of hirudin significantly inhibited hydrogen peroxide-induced β-galactosidase production associated with fibroblast senescence (Figure 4C). Q-PCR results also revealed that 10 µΜ and 30 µΜ of hirudin effectively reduced the mRNA expression levels of IL-6, IL-1β, TNF-α, and IL-8 (Figure 4D–G). We also found that hirudin at concentrations of 10 µΜ and 30 µΜ effectively reduced the expression levels of the cellular senescence markers p21 and p16 (Figure 4H–K). These results indicated that hirudin could significantly inhibit fibroblast senescence, with the 30 µΜ dosage having a more significant effect than that of the 10 µΜ dosage.

### 3.5. PGC1-Alpha/Sirt3 Pathway Mediated the Anti-Fibroblast Senescence Effect of Hirudin

**Figure 5:**
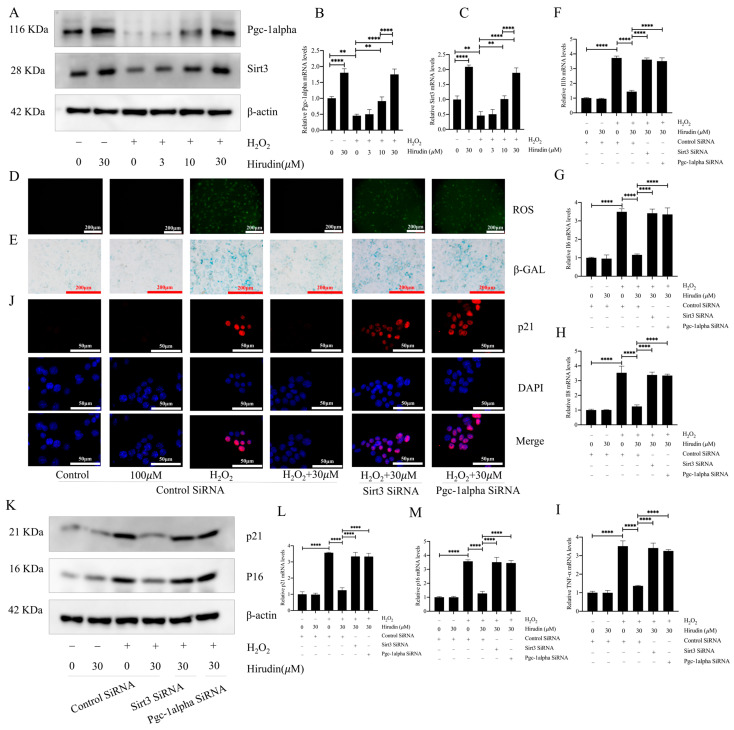
Hirudin activated the PGC1-alpha/Sirt3 pathway to inhibit fibroblast senescence. The protein expression levels of PGC1-alpha and Sirt3 in fibroblasts (**A**). The mRNA expression levels of *PGC1-alpha* and *Sirt3* in fibroblasts (**B**,**C**). The levels of ROS in fibroblasts (**D**). β-galactosidase levels in fibroblasts (**E**). The mRNA expression levels of *IL1b*, *TNF-α*, *IL8*, and *IL6* in fibroblasts (**F**–**I**). The expression of p21 in fibroblasts (**J**). The protein expression levels of p21 and p16 in fibroblasts (**K**). The mRNA expression levels of *p21* and *p16* in fibroblasts (**L**,**M**). All data are expressed in the mean ± standard deviation, and all experiments were conducted independently and repeated a minimum of three times. ** *p* < 0.01; **** *p* < 0.0001.

To further investigate the anti-fibroblast senescence mechanism of hirudin, we examined its effect on PGC1-alpha and Sirt3 in fibroblasts. This was considered due to the significant elevation in PGC1-alpha and Sirt3 expression levels in the lung tissues of mice with pulmonary fibrosis upon hirudin treatment, and the important role of the PGC1-alpha/Sirt3 pathway in senescence. The findings demonstrated that 10 µΜ and 30 µΜ concentrations of hirudin notably increased the protein and mRNA expression levels of PGC1-alpha and Sirt3 (Figure 5A–C). The effects of hirudin on the content of ROS and β-galactosidase, the expression levels of p16 and p21, and the expression level of SASPs during fibroblast senescence were suppressed after the specific silencing of genes associated with PGC1-alpha and Sirt3, respectively (Figure 5D–M). Thus, the inhibitory effect of hirudin on fibroblast senescence was mediated by the PGC1-alpha/Sirt3 pathway.

### 3.6. Schematic of a Model of the Anti-Pulmonary Fibrosis Effect of Hirudin

**Figure 6:**
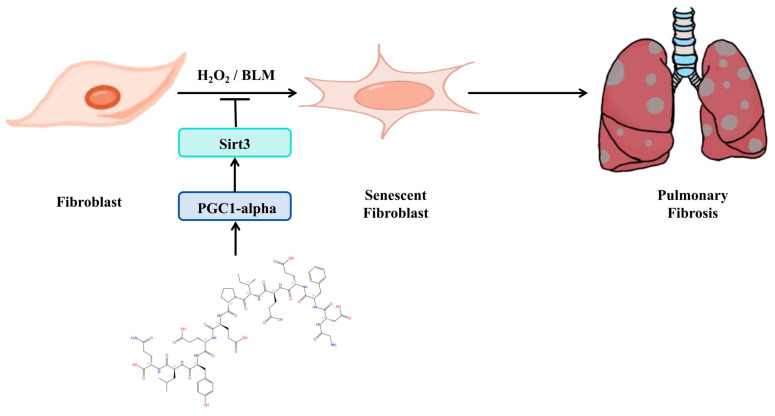
Schematic of a model of the anti-pulmonary fibrosis effect of hirudin.

In summary (Figure 6), hirudin inhibited fibroblast senescence through activation of the PGC1-alpha/Sirt3 pathway, which in turn exerted its anti-pulmonary fibrosis effect.

## 4. Discussion

This study is the first to demonstrate that hirudin inhibits fibroblast senescence by exerting an anti-oxidative stress effect through activation of the PGC1-alpha/Sirt3 pathway, thereby suppressing pulmonary fibrosis. This study not only expands on the pharmacological effects of hirudin but also establishes a novel experimental basis for the pharmacological treatment of IPF. It suggests that activating the PGC1-alpha/Sirt3 pathway could represent a novel target for the inhibition of fibroblast senescence in IPF.

After intratracheal injection of bleomycin, mice generally underwent the following phases: Acute lung injury and inflammation occurred from day 1 to day 7, during which the number of inflammatory cells increased and multiple inflammatory mediators were activated. Inflammation gradually subsided from days 7 to 14; however, the inflammatory response continued to be predominant pathological feature of this phase. After day 14, myofibroblast numbers and ECM deposition increased, with collagen and ECM deposition peaking by day 28 [32]. Therefore, the initiation of pharmacological interventions between days 1 and 14 after modeling cannot exclude the influence of pharmacological anti-inflammatory effects on pulmonary fibrosis [33]. In contrast to the study conducted by Shen et al. [25], which investigated the therapeutic effect of rhSOD-hirudin on pulmonary fibrosis by administering rhSOD-hirudin starting on day 3 or 7 after tracheal injection of bleomycin, our study focused on elucidating the direct anti-pulmonary fibrosis effect and its associated mechanisms. This was achieved through intraperitoneal injection of varying concentrations of hirudin on days 15–28. The results showed that hirudin significantly improved the disorganization of the lung tissue structure and reduced the expression levels of lung fibrosis-related indices in mice with pulmonary fibrosis, indicating that hirudin had a direct anti-fibrotic effect on lung fibrosis. However, we did not compare the differences in the therapeutic effects of hirudin on pulmonary fibrosis with those of drugs currently used clinically for the treatment of IPF, which warrants further exploration in future studies. In addition, not thoroughly exploring the potential toxicity or side effects of high-dose hirudin is where the inadequacy of our experimental design lies, and we will also further explore the detailed dose–response and potential side effects in future studies to provide a more solid background foundation for the clinical application of hirudin.

Oxidative stress significantly contributes to both cellular senescence and the development of IPF [34,35,36,37]. ROS may drive cellular senescence through DNA damage and activation of DNA damage-response-associated pathways, and the senescence of a variety of cells, including fibroblasts, leads to the progression of IPF. Moreover, mitochondrial dysfunction in senescent cells leads to increased ROS production, which may further contribute to the progression of cellular senescence and fibrosis [38]. In our study, we found that hirudin was effective in lowering the levels of ROS and multiple markers of oxidative stress and in inhibiting fibroblast senescence while exerting anti-pulmonary fibrosis effects. In addition to fibroblast senescence, the senescence of other key cells within lung tissue, such as epithelial cells and macrophages, has been recognized as significant in the progression of IPF [6]. Notably, whether hirudin exhibits an inhibitory effect on the senescence of cells beyond fibroblasts has not been investigated in our study, presenting an area for further refinement and exploration.

In exploring the specific mechanism by which hirudin exerts anti-oxidative stress and inhibits fibroblast senescence to exert anti-pulmonary fibrosis effects, we found that hirudin promoted the expression of PGC1-alpha and Sirt3 in the lung tissues of mice with pulmonary fibrosis and fibroblasts. Moreover, the anti-oxidative stress and anti-fibroblast senescence effects of hirudin were suppressed after the specific silencing of genes associated with PGC1-alpha or Sirt3 using siRNA in vitro. This suggested that the PGC1-alpha/Sirt3 pathway mediates the inhibitory effect of hirudin on fibroblast senescence-induced pulmonary fibrosis and anti-oxidative stress. In addition to Sirt3, other members of the sirtuin family, such as Sirt1 and Sirt6, have demonstrated anti-pulmonary fibrosis effects through the inhibition of cellular senescence [39]. Therefore, it is still worthwhile to further investigate whether hirudin has a promotional effect on the expression of other sirtuin family members, including Sirt1 and Sirt6. Additionally, the PGC1-alpha/Sirt3 pathway plays an important role in the regulation of mitochondrial function [40]; hirudin may have inhibited fibroblast senescence and thus exerted its anti-fibrotic effect by regulating mitochondrial function through the PGC-alpha/Sirt3 pathway. This will be explored further in future studies.

Thrombin, as a modulator of inflammatory and reparative processes in tissues, has been shown to promote the development of a variety of diseases, including pulmonary fibrosis [41,42,43]. Moreover, thrombin has been shown to promote cellular senescence [44]. In our study, the potent thrombin inhibitor, hirudin, inhibited the development of pulmonary fibrosis by inhibiting fibroblast senescence. In addition, in recent years, hirudin has been used to prevent and treat various diseases, including chronic kidney disease, stroke, and renal fibrosis [45,46,47]. Therefore, whether the therapeutic effects of hirudin on these diseases are also related to its anti-senescence effects and the potential link between the multiple pharmacological effects of hirudin deserve further investigation. Moreover, this study is the first to propose the PGC1-alpha/Sirt3 pathway as a novel drug target for the treatment of pulmonary fibrosis by inhibiting fibroblast senescence. This proposition has important theoretical value as it reveals a novel molecular mechanism for the treatment of pulmonary fibrosis. Furthermore, from a clinical perspective, targeting the PGC1-alpha/Sirt3 pathway may enable the development of innovative therapeutic strategies for the treatment of pulmonary fibrosis, which could help address the critical medical need for pulmonary fibrosis therapy.

## 5. Conclusions

By observing the inhibitory effect of hirudin on fibroblast senescence, we elucidated how it exerts its anti-fibrotic effects. Hirudin inhibited fibroblast senescence by activating the PGC1-alpha/Sirt3 pathway through promoting the expression of PGC1-alpha and Sirt3; that is the specific mechanism by which it exerts its anti-pulmonary fibrosis effect. Our results suggest that hirudin may be a therapeutic agent for pulmonary fibrosis, and we propose that the PGC1-alpha/Sirt3 pathway may be a potential therapeutic target for pulmonary fibrosis.

## Figures and Tables

**Table 1:** Sequences.

Gene Name	Forward [5′-3′]	Reverse [5′-3′]
Mouse *col1a1*	GAGCGGAGAGTACTGGATCG	GCTTCTTTTCCTTGGGGTTC
Mouse *α-SMA*	TGGCTATTCAGGCTGTGCTGTC	CAATCTCACGCTCGGCAGTAGT
Mouse *β-actin*	GTGCTATGTTGCTCTAGACTTCG	ATGCCACAGGATTCCATACC
Mouse *p21*	CCTGGTGATGTCCGACCTGTTC	CGAAGTCAAAGTTCCACCGTTCTC
Mouse *p16*	AAGAGCGGGGACATCAAGACATC	AAAGACCACCCAGCGGAACAC
Mouse *PGC1-alpha*	TTCGCTGCTCTTGAGAATGGATATAC	TCGTCTGAGTTGGTATCTAGGTCTG
Mouse *Sirt3*	CTCTACAGCAACCTTCAGCAGTATG	CAGGAAGTAGTGAGTGACATTGGG
Mouse *TNF-α*	GTGCCTATGTCTCAGCCTCTTCTC	TGGTTTGTGAGTGTGAGGGTCTG
Mouse *IL1b*	CTCGCAGCAGCACATCAACAAG	CCACGGGAAAGACACAGGTAGC
Mouse *IL6*	TTCTTGGGACTGATGCTGGTGAC	GTGGTATCCTCTGTGAAGTCTCCTC
Mouse *IL8*	CTCCTGCTGGCTGTCCTTAACC	TGGGACTGCTATCACTTCCTTTCTG

## Data Availability

Any necessary data from this study can be obtained by contacting the corresponding authors.
